# Comparing Effects of Tolvaptan and Instruction to Increase Water Consumption in ADPKD: *Post Hoc* Analysis of TEMPO 3:4

**DOI:** 10.34067/KID.0000000000000302

**Published:** 2023-11-21

**Authors:** Joga Gobburu, Vijay Ivaturi, Xiaofeng Wang, Susan E. Shoaf, Pravin Jadhav, Ronald D. Perrone

**Affiliations:** 1 Pumas-AI, Baltimore, Maryland; 2 Otsuka Pharmaceutical Development & Commercialization (OPDC), Inc., Princeton, New Jersey; 3 MDCI Biosciences LLC, Philadelphia, Pennsylvania; 4 Division of Nephrology, Tufts Medical Center, Boston, Massachusetts

**Keywords:** ADPKD, CKD, clinical nephrology, clinical trial, genetic kidney disease, osmolality, polycystic kidney disease

## Abstract

**Key Points:**

- In a *post hoc* analysis, short-term reduction in spot urine osmolality (Uosm) was associated with decreased kidney volume growth in autosomal dominant polycystic kidney disease for both tolvaptan and instruction to increase hydration alone.
- For the same spot Uosm reduction, however, the kidney volume benefit was greater with tolvaptan, possibly because of greater cumulative 24-hour Uosm suppression by tolvaptan.

**Background:**

In addition to decreasing water excretion and increasing urinary concentration, the antidiuretic hormone vasopressin plays a role in the pathophysiology of autosomal dominant polycystic kidney disease. It has been hypothesized that by suppressing vasopressin release, drinking large amounts of water might exert therapeutic effects in autosomal dominant polycystic kidney disease similar to those of tolvaptan, an antagonist of the vasopressin type 2 receptor, but evidence is lacking. We analyzed data from tolvaptan clinical trials to evaluate relationships among water intake, urine osmolality (Uosm), and change in total kidney volume (TKV).

**Methods:**

Analysis of the Tolvaptan Efficacy and Safety in Management of Autosomal Dominant Polycystic Kidney Disease and Its Outcomes 3:4 clinical trial in which participants were randomized to tolvaptan or placebo and instructed to drink large amounts of water. The relationship between change in spot Uosm from baseline to week 3 and change in TKV to month 12 was assessed using linear regression modeling. Two short-term tolvaptan trials were analyzed to explore relationships between intermittent Uosm sampling and 24-hour Uosm suppression.

**Results:**

With both tolvaptan and placebo (*i.e.*, mandated high water intake alone), Uosm reduction at week 3 was associated with reduction in TKV growth at month 12. However, for the same decrease in spot Uosm, the corresponding reduction in TKV growth was greater for tolvaptan (*e.g.*, a −250 mOsm/kg reduction in Uosm at week 3 was associated with a −1% change in TKV at month 12 for tolvaptan versus +4.5% for placebo). In short-term trials, similar reductions in spot or trough Uosm values were achievable with tolvaptan and high water intake, but cumulative 24-hour suppression was greater with tolvaptan.

**Conclusions:**

This analysis supports a relationship between effects on Uosm and inhibition of disease progression by tolvaptan and high water intake alone. The findings further suggest that 24-hour Uosm measurement is superior to spot Uosm for assessing suppression of vasopressin activity by tolvaptan.

## Graphical abstract

**Figure:**
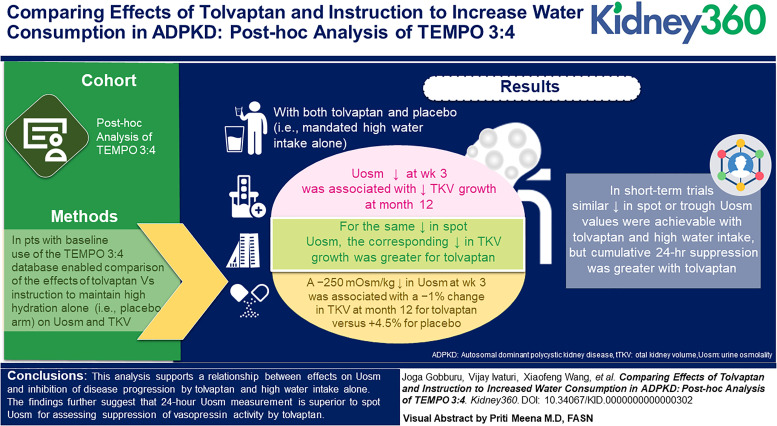

## Introduction

An important physiologic function of the antidiuretic hormone arginine vasopressin is to increase water reabsorption in the renal collecting ducts, reducing water excretion by the kidneys and increasing urine osmolality (Uosm). Activation of the vasopressin type 2 receptor (V2R) on epithelial cells of the collecting duct triggers a cyclic adenosine monophosphate–mediated intracellular signaling cascade that results in the insertion of aquaporin-2 water channels into the apical plasma membrane. The collecting ducts become water permeable, effecting osmotic equilibration of the tubular fluid with the surrounding hypertonic medullary interstitium.^1^

Dysregulation of vasopressin activity plays an important role in the pathophysiology of autosomal dominant polycystic kidney disease (ADPKD). Activation of V2R has been shown to stimulate renal epithelial cell proliferation (cyst formation) and fluid secretion into cysts through cyclic adenosine monophosphate–dependent mechanisms.^2^

Inhibition of vasopressin action by the V2R antagonist tolvaptan exerts opposite effects, resulting in decreases in Uosm (hypotonic urine) and slowing of cyst volume expansion.^3,4^ Because the effects of vasopressin or tolvaptan in both collecting ducts and kidney cysts are mediated by the same V2R, their physiologic effects in each system are mirrored by those in the other. The efficacy of tolvaptan in slowing rates of kidney cystic expansion and eGFR decline has been demonstrated in clinical trials.^4,5^

On the basis of observations about vasopressin in ADPKD progression, it has been proposed that increased water intake by patients with ADPKD may exert similar effects in slowing disease progression because vasopressin release is driven by plasma osmolality and is suppressed by drinking beyond thirst.^6^ Short-term (≤8 week) studies in patients with ADPKD have demonstrated that patients can adhere to high water intake, thereby lowering Uosm and levels of copeptin, a marker of vasopressin.^7–10^ Data on associations of increased water intake and low osmolar diet with ADPKD-related outcomes for a longer period have, until recently, been lacking. The PREVENT-ADPKD trial, a 3-year trial evaluating effects of high water intake, failed to show benefits for total kidney volume (TKV), eGFR, and other end points.^11,12^ Only 52% of the participants assigned to high water intake achieved the target Uosm of 270 mOsmol/kg.^12^ There were no differences in TKV progression or serum copeptin in this trial between the high or ad libitum water intake groups. Because almost half of the participants assigned to high intake failed to achieve the targeted Uosm, this trial calls into question whether the strategy of high water intake alone is adequate to suppress vasopressin secretion.

Questions about the relationships of water intake, Uosm, and vasopressin levels to ADPKD progression require further research. Uosm varies widely over the course of the day, changing in response to water intake and periods of water deprivation (*e.g.*, during sleep).^1^ In studies of high water intake in ADPKD, Uosm has been measured as a spot value^7,10^ or a mean value derived from 24-hour urine collection.^8,9,12^ In long-term tolvaptan clinical trials, patients with ADPKD received tolvaptan or placebo, with participants in all study treatment arms encouraged to maintain large water intake,^4,5^ and participants were assessed for Uosm in various schedules in different studies. Accordingly, tolvaptan clinical trials provide a dataset that enables assessment of the effects of tolvaptan versus recommended high water intake alone on Uosm.

We analyzed data from the 3-year Tolvaptan Efficacy and Safety in Management of Autosomal Dominant Polycystic Kidney Disease and Its Outcomes (TEMPO) 3:4 trial^4^ and two short-term trials^3,13^ to compare tolvaptan and recommended high water intake for their effects on Uosm and how short-term changes in Uosm may have been related to longer-term effects on TKV.

## Methods

Data were analyzed from three clinical trials of tolvaptan conducted in adults with ADPKD, including a phase 3, randomized, double-blind, placebo-controlled, 3-year trial (TEMPO 3:4 [NCT00428948]) and two short-term, phase 2 trials.^4^ The short-term trials were trial 156-04-248 (trial 248), which evaluated single ascending doses of tolvaptan in 11 participants,^3^ and trial 156-09-285 (NCT01210560; trial 285), a randomized, double-blind, placebo-controlled, dose-ranging study in 25 participants that consisted of three crossover treatment periods of 7 days each.^13^

Use of the TEMPO 3:4 database enabled comparison of the effects of tolvaptan versus instruction to maintain high hydration alone (*i.e.*, placebo arm) on Uosm and TKV in a large study population. By enrollment criteria, participants (tolvaptan, *n*=961; placebo, *n*=483) at baseline had largely intact kidney function (estimated creatinine clearance ≥60 ml/min) with a high likelihood of rapid disease progression (TKV ≥750 ml). Per study protocol, nonfasting spot Uosm was assessed immediately before morning dosing from a second urine void taken after the first morning's void, ideally as a midstream, clean catch sample.

The percentage change from baseline in TKV at month 12 was regressed against changes from baseline in morning predose spot Uosm at week 3. The week 3 time point for Uosm was chosen as the principal marker because it reflects the full effect of tolvaptan on this biomarker. The relationship was evaluated using graphical and regression analyses. Each patient contributed one pair of values for change from baseline in spot Uosm at week 3 and percentage change from baseline in TKV at month 12.

The proportion of tolvaptan treatment effect on TKV that was attributable to changes in Uosm was estimated in an analysis performed in three sequential steps: (*1*) determine the magnitude of the treatment effect on change in TKV from baseline to month 12 using regression analysis with treatment as covariate; (*2*) test whether including change in Uosm to week 3 in the model modified the treatment effect; and (*3*) compute the proportion of the tolvaptan treatment effect on change in TKV to month 12 explained by change in Uosm to week 3 when treatment is removed from the model.

Whereas only spot Uosm (effect at the lowest tolvaptan concentration) was collected in TEMPO 3:4, more extensive Uosm data were available from the short-term trials. In the present analysis, data from trial 248 were plotted to explore the relationship between trough Uosm (*i.e.*, 16–24 hours after dosing) and 24-hour area under the Uosm time curve (AUC_24_) for tolvaptan and placebo. Data from trial 285 were plotted to explore the relationship between spot Uosm and Uosm AUC_24_ for tolvaptan and placebo. For the analyses of both trials, baseline (pretreatment) Uosm values from the tolvaptan treatment groups were included with values from the placebo groups as placebo data in graphs.

## Results

**Figure 1:**
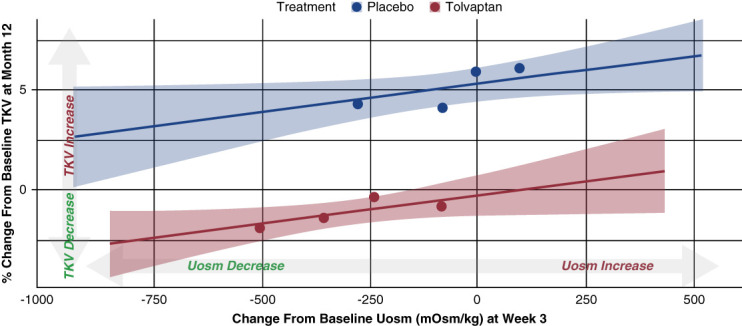
**Relationship between changes in spot Uosm at week 3 and percentage changes in TKV at month 12 for placebo (mandated high water intake alone) and tolvaptan in TEMPO 3:4.** The graph represents a linear regression line and 95% CI using participant-level data. The quartiles are overlaid to represent trends in the raw data. CI, confidence interval; TEMPO, Tolvaptan Efficacy and Safety in Management of Autosomal Dominant Polycystic Kidney Disease and Its Outcomes; TKV, total kidney volume; Uosm, urine osmolality.

Analysis of the relationship between change from baseline in spot Uosm and percentage change from baseline in TKV in TEMPO 3:4 indicated that for both tolvaptan and placebo (recommended high water intake only), a greater decrease in Uosm at week 3 was associated with lesser TKV growth at month 12 (Figure 1). However, for the same level of spot Uosm reduction, the corresponding TKV growth reduction was greater for tolvaptan than placebo. For example, a −250 mOsm/kg change in Uosm at week 3 was associated with a −1% change in TKV at month 12 for tolvaptan versus +4.5% for placebo.

The three-step analysis to estimate the proportion of the tolvaptan treatment effect on TKV that was attributable to change in Uosm yielded the following results. In regression modeling using treatment as a covariate, tolvaptan significantly reduced growth in TKV from baseline to month 12 compared with placebo (−6.31%; *P* < 0.001). Age, sex, race, weight, baseline eGFR, end of treatment eGFR, baseline TKV, baseline Uosm, change in Uosm to week 3, and treatment were tested individually as covariates in the regression model to identify significant predictors of treatment effect. Excluding factors that were highly correlated with other factors, the covariates retained in the final model were age, sex, change in Uosm to week 3, and treatment. Inclusion of change in Uosm to week 3 in the multivariate model reduced the tolvaptan treatment effect on TKV from −6.31% to −5.37% (Table 1). Change in spot Uosm at week 3 therefore explained 14.9% of the tolvaptan treatment effect on TKV (100%×[1–5.37/6.31]).

**Table 1:** Results of a multivariate regression analysis of tolvaptan treatment effect on change in total kidney volume to month 12 in TEMPO 3:4 with inclusion of change from baseline to week 3 in urine osmolality as a covariate

Parameter	Estimate	95% CI	*P* Value
Intercept, %	17.01	13.86 to 20.17	<0.00001
ΔUosm3, mOsm/kg	0.0041	0.0014 to 0.0067	0.00220
Age, yr	−0.158	−0.228 to −0.088	<0.00001
Sex (one, male; two, female)	−3.672	−4.647 to −2.697	<0.00001
**Model with treatment and the above predictors (0, placebo; 1, tolvaptan)**			
Treatment, %	−5.37	−6.55 to −4.18	<0.00001

CI, confidence interval; TEMPO, Tolvaptan Efficacy and Safety in Management of Autosomal Dominant Polycystic Kidney Disease and Its Outcomes; TKV, total kidney volume; ΔUosm3, change from baseline to week 3 in urine osmolality.

**Figure 2:**
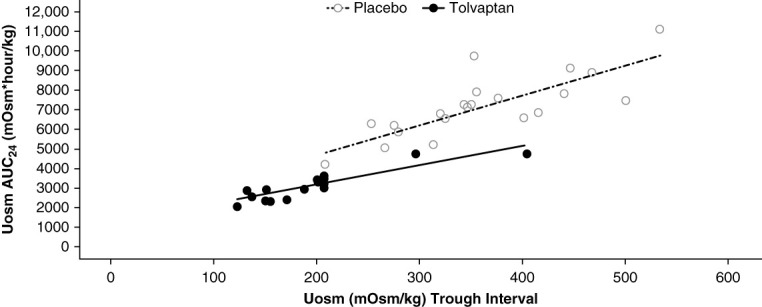
**Relationship between Uosm at the trough interval (16–24 hours postdose) and Uosm AUC**_**24**_ **for placebo and tolvaptan in trial 248.** Data are shown for all participants from pretreatment baseline and placebo on other occasions (hollow circles) and for participants who received 60 or 120 mg tolvaptan (filled circles). Similar trough values for tolvaptan and placebo in the presence of lower AUC_24_ for tolvaptan were driven by higher 24-hour urine volume with tolvaptan. AUC_24_, 24-hour area under the Uosm time curve.

Relationships between Uosm values at discrete time points (Uosm during the trough interval or spot Uosm) and Uosm AUC_24_ were assessed for the short-term trials. In trial 248, similar trough Uosm values for tolvaptan and placebo were associated with lower AUC_24_ with tolvaptan compared with placebo (Figure 2). A trough Uosm of 300 mOsm/kg, for example, reflected a Uosm AUC_24_ (in mOsm×h/kg) of approximately 6180 for placebo and approximately 4180 for tolvaptan. Stated differently, achievement of the same Uosm trough value was associated with greater cumulative 24-hour suppression of Uosm with tolvaptan compared with placebo. Similar trough Uosm values for tolvaptan and placebo in the presence of lower AUC_24_ for tolvaptan were driven by higher 24-hour urine volume with tolvaptan.

**Figure 3:**
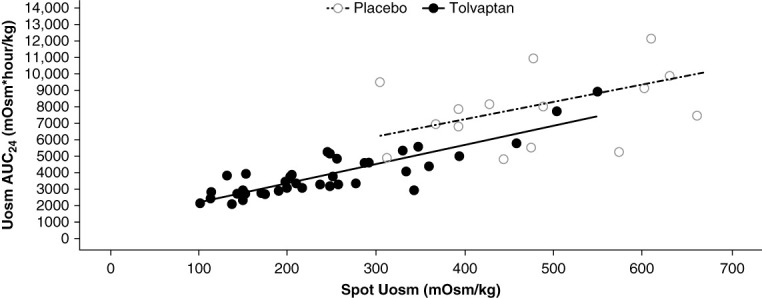
**Relationship between spot Uosm and Uosm AUC**_**24**_ **for placebo and tolvaptan in trial 285.** Data are shown for participants at baseline (hollow circles) and for participants who received MR 120 mg, MR 60 mg, or IR 90+30 mg tolvaptan (filled circles). Similar spot values for tolvaptan and placebo in the presence of lower AUC_24_ for tolvaptan were driven by higher 24-hour urine volume with tolvaptan. IR, immediate release; MR, modified release.

The results were similar in trial 285 for the spot Uosm–AUC_24_ relationship (Figure 3). For the same spot value, AUC_24_ was less with tolvaptan than placebo, again driven by higher urine output with tolvaptan.

## Discussion

This *post hoc* analysis of data from TEMPO 3:4 supports reduction in Uosm as an indicator of longer-term effects on TKV during tolvaptan V2R antagonist therapy, a relationship with a mechanistic basis in the dual role of vasopressin in increasing urinary concentration and driving cystic proliferation and expansion in ADPKD.^2^ The observation that tolvaptan was associated with greater inhibition of TKV growth in TEMPO 3:4 at month 12 than placebo for similar spot Uosm values at week 3 suggests the limitations of spot Uosm as a means of measuring the suppression of vasopressin activity. These limitations are underscored by the low proportion of tolvaptan treatment effect explained by change in spot Uosm (15%).

The differences between spot and 24-hour measures of Uosm shown here help explain the previous finding that in TEMPO 3:4, greater Uosm suppression was significantly associated with slower kidney function decline over 3 years in tolvaptan-treated participants, but not in placebo-treated participants.^14^ As in that earlier analysis, the relationship between Uosm and treatment outcome (*i.e.*, TKV) in our analysis may have been different between tolvaptan and placebo because 24-hour effects of tolvaptan on Uosm were not fully captured by the Uosm measurement methodology used in TEMPO 3:4.

Given the variability of vasopressin levels throughout a 24-hour period,^1^ 24-hour measures of Uosm are most appropriate to assess the effects of therapeutic interventions on Uosm. This analysis of TEMPO 3:4 data indicates that instruction to maintain high hydration can achieve similar spot Uosm values as tolvaptan therapy, but consistent suppression of antidiuretic hormone through water intake during daytime and sleep periods would be difficult to achieve. The daily split dosing regimen for tolvaptan, with a larger dose in the morning and a smaller dose 8 hours later, is designed to produce sustained 24-hour suppression of vasopressin activity with minimized nocturia.^15^ Trials of dietary modification and increased water intake have indicated the short-term feasibility of such regimens in patients with ADPKD, but participants were assessed for Uosm using spot values^7,10^ or a mean value obtained by 24-hour urine collection.^8,9^ Our analyses of short-term tolvaptan clinical trials in ADPKD support the distinction between Uosm values at specific time points and cumulative daily suppression, with tolvaptan-treated patients exhibiting lower Uosm AUC_24_ than placebo-treated patients at similar spot or trough Uosm.

This analysis is limited by its retrospective nature. Prospective, 3-year data on the effects of increased water intake in ADPKD demonstrated that not all individuals can maintain a sufficiently high water intake to substantially suppress vasopressin and that inadequate suppression does not improve ADPKD outcomes.^12^ The findings presented here, in conjunction with earlier data,^14^ support the value of consistent suppression of vasopressin activity to minimize disease progression.

## Data Availability

Anonymized data created for the study are or will be available in a persistent repository on publication. Analyzable Data. Clinical Trial Data. To submit inquiries related to Otsuka clinical research, or to request access to individual participant data (IPD) associated with any Otsuka clinical trial, please visit https://clinical-trials.otsuka.com/. For all approved IPD access requests, Otsuka will share anonymized IPD on a remotely accessible data sharing platform. https://clinical-trials.otsuka.com/.
